# Supplementary figures and images for: A divergent CheW confers plasticity to nucleoid-associated chemosensory arrays
Source: PLoS Genet. 2019 Dec 20;15(12):e1008533. doi: 10.1371/journal.pgen.1008533 (PMC6952110; doi:10.1371/journal.pgen.1008533)

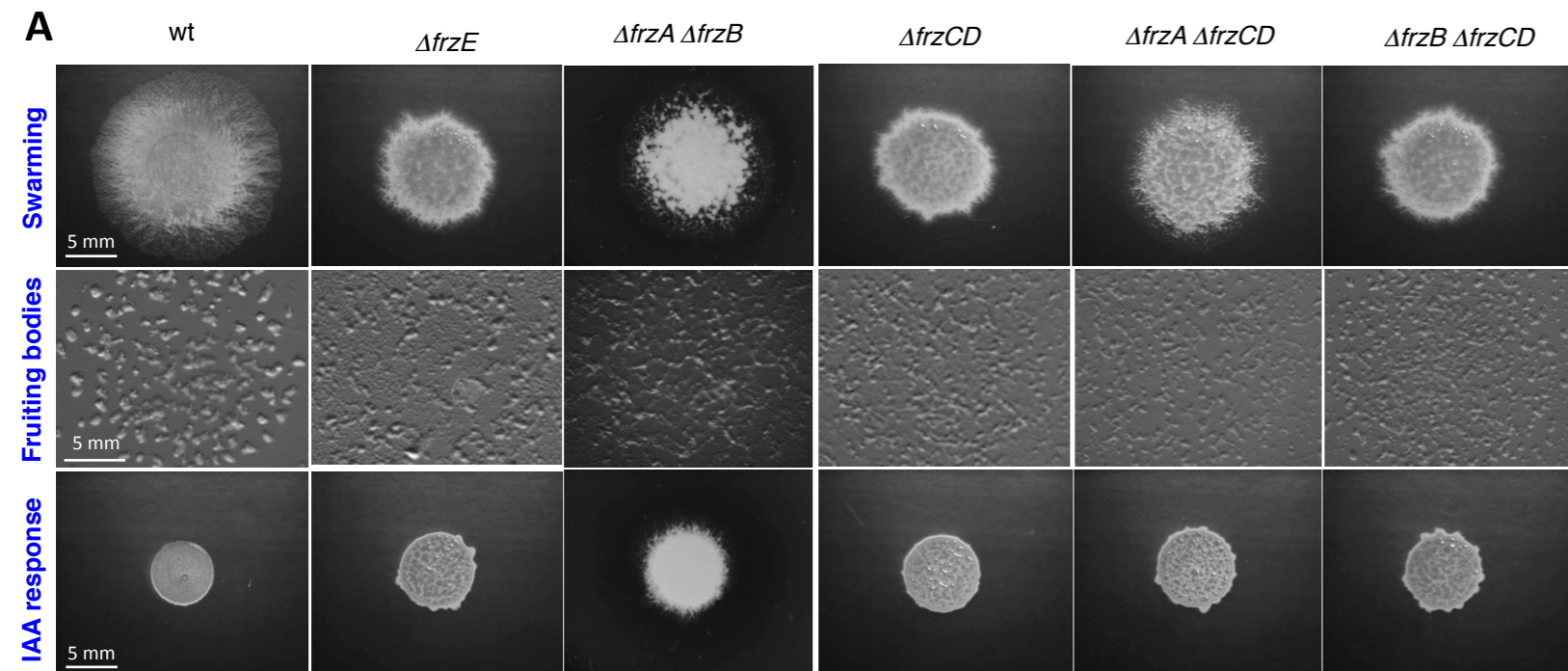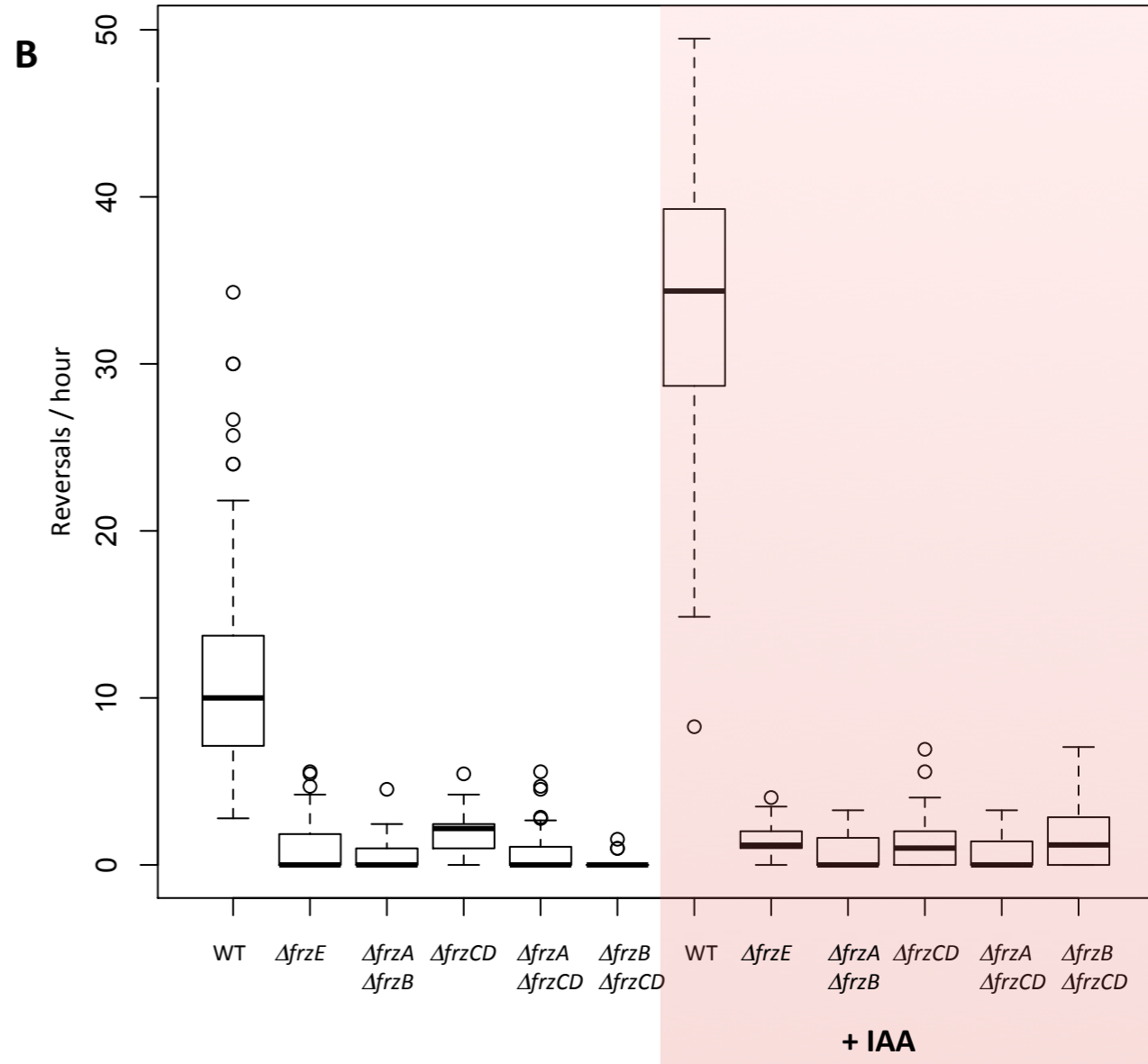

Supplement: S1 Fig — (A) Motility and fruiting body formation phenotypes were photographed at 48h and 72h, respectively. (B) Box plots of reversal frequencies of single cells moving on agar pads supplemented or not with 0.15% IAA. The lower and upper boundaries of the boxes correspond to 25% and 75% percentiles, respectively. The median is shown as a line at the center of each box and whiskers represents the 10% and 90% percentiles. For the reversal frequency measurements, cells issued from two biological replicates, were used. For WT approximately 500 cells, issued from six biological replicates, were used. (PDF) [file pgen.1008533.s001.pdf]

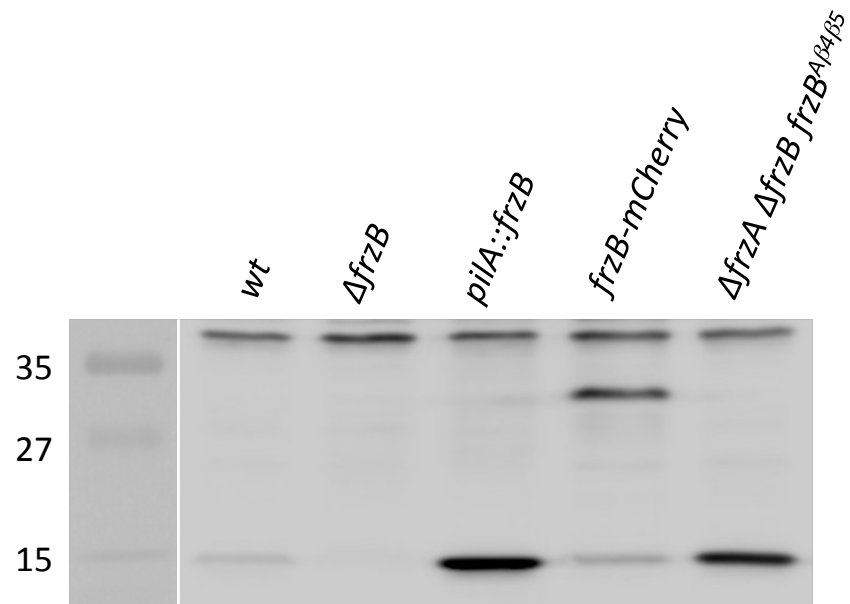

Supplement: S2 Fig — The pilA::frzB strain, used as positive control, expresses frzB under the control of the pilA promoter for high expression. The white line is used to indicate that two lanes from the same gel were separated by other lanes in the original gel. (PDF) [file pgen.1008533.s002.pdf]

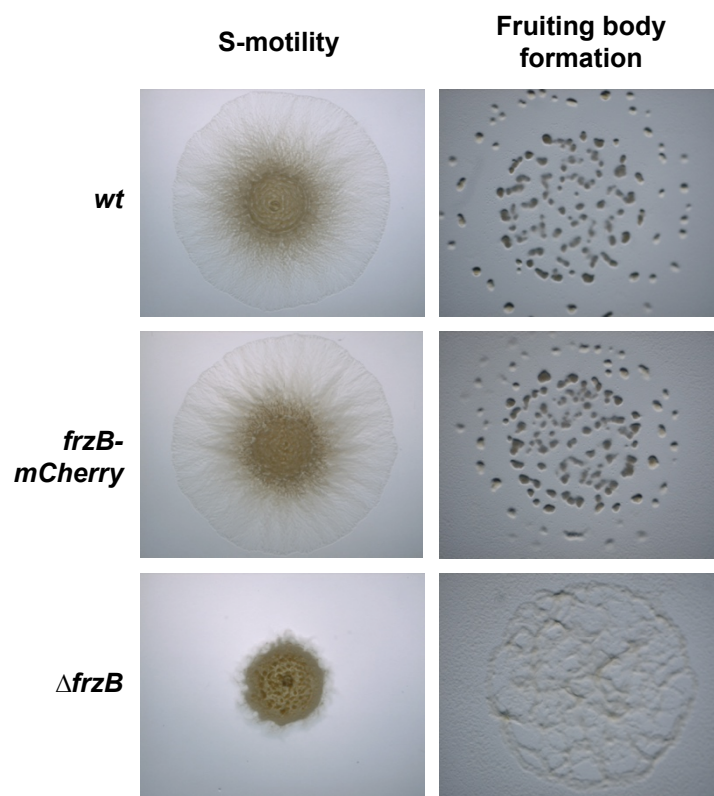

Figure S3

Supplement: S3 Fig — (PDF) [file pgen.1008533.s003.pdf]

$\Delta frzA \Delta frzB$   
 $frzB^{84\beta 5}$

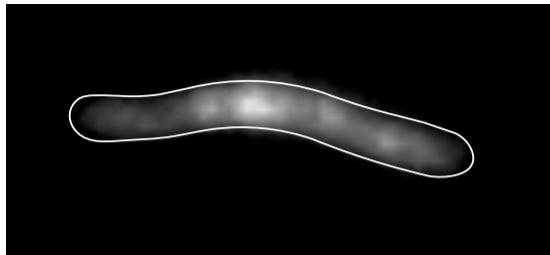

$\Delta frzA \Delta frzB$

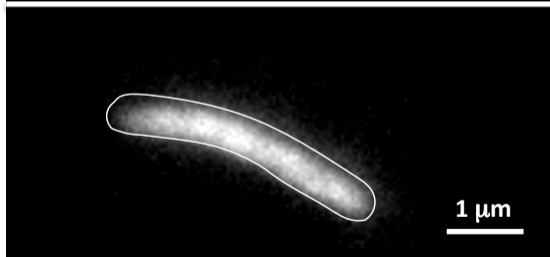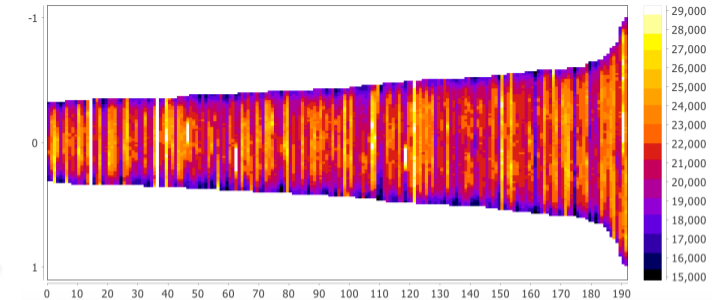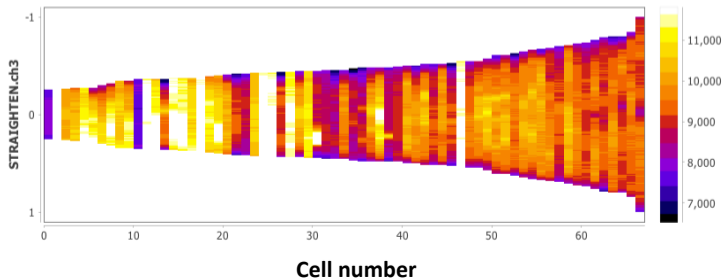

Supplement: S4 Fig — (Left) Fluorescence micrographs of the indicated M. xanthus strains carrying FrzCD-gfp fusions. The cell boundaries were drawn manually from the phase-contrast images. (Right) For each indicated strain, more than 120 cells (x axis) from at least two biological replicates are represented as lines and ordered according to their length (pixels) in demographs. The GFP fluorescence intensity along the cell body is represented as colored pixels at the corresponding cell position (from -1 to +1 on the y axis). “0” is the cell center. On the right, a scale indicates the fluorescence intensity and the corresponding colors. (PDF) [file pgen.1008533.s004.pdf]

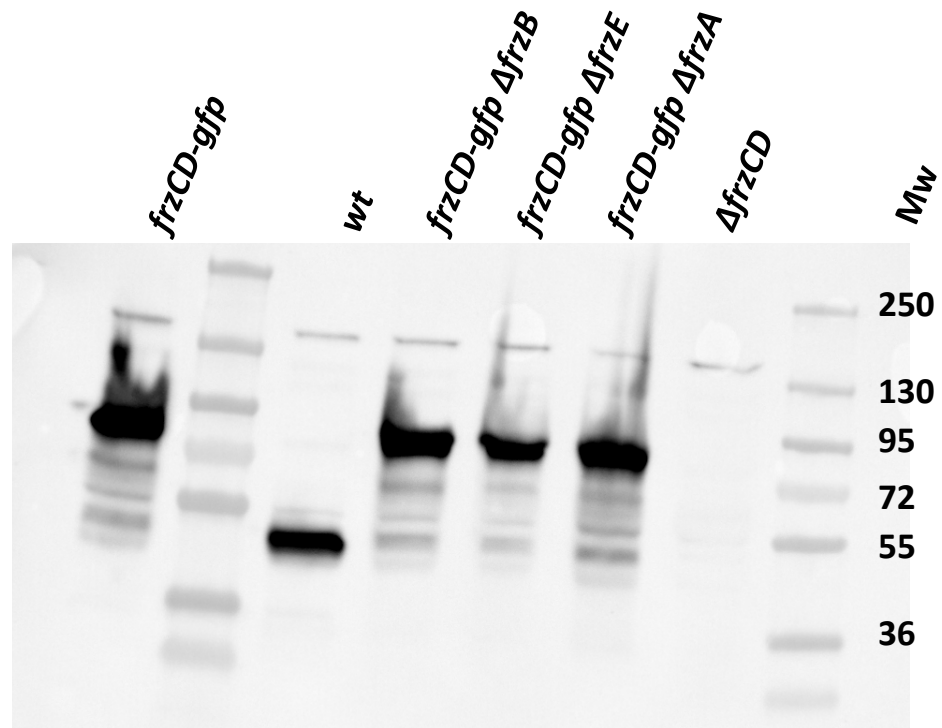

Supplement: S5 Fig — (PDF) [file pgen.1008533.s005.pdf]

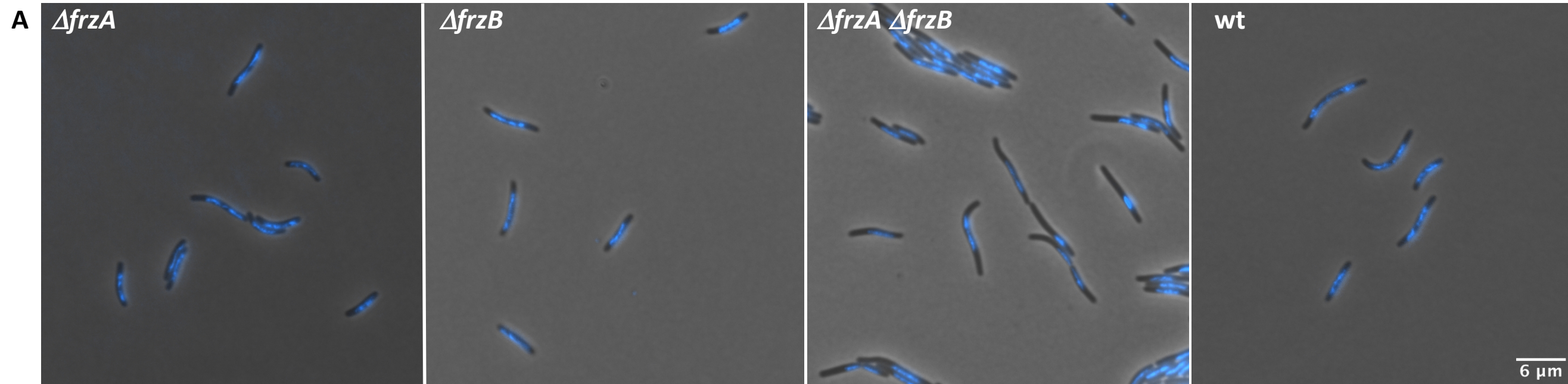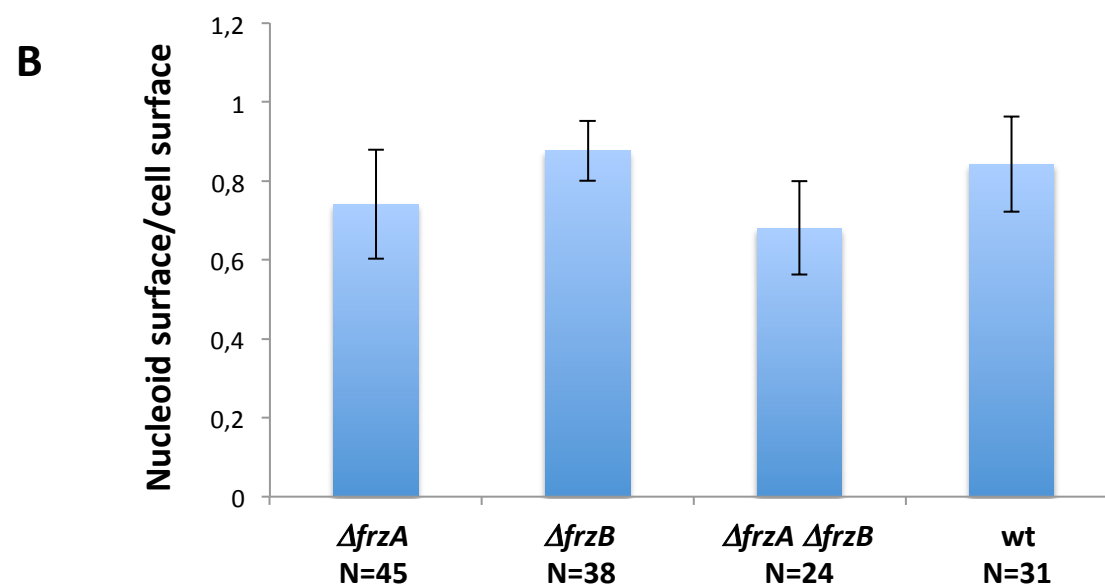

Supplement: S6 Fig — (A) Cells of the indicated strains were incubated 30 minutes with DAPI and then imaged at the fluorescence microscope. The nucleoid and cell surfaces were measured automatically with Microbe J [44]. (B) The ratio between the nucleoid and cell surface was then calculated for each cell and averages values plotted. The numbers of analyzed cells are indicated per each strain. Cells were issued from two independent biological replicates. (PDF) [file pgen.1008533.s006.pdf]
