# Supplementary material for: A divergent CheW confers plasticity to nucleoid-associated chemosensory arrays
Source: PLoS Genet. 2019 Dec 20;15(12):e1008533. doi: 10.1371/journal.pgen.1008533 (PMC6952110; doi:10.1371/journal.pgen.1008533)
Supplement: S1 Table — (PDF) [file pgen.1008533.s007.pdf]

Table S1. Strain list

Strains	Genotype	Reference or source
DZ2	*wt*	Zusman et al., 1982
DZ4478	*ΔfrzA*	Bustamante et al ; 2004
DZ4479	*ΔfrzB*	Bustamante et al ; 2004
DZ4487	*frzCDΔ6-182*	Bustamante et al ; 2004
DZ4480	*ΔfrzCD*	Bustamante et al ; 2004
EM538	*ΔfrzA frzCDΔ6-182*	This study
EM539	*ΔfrzB frzCDΔ6-182*	This study
DZ4620	*frzCD-gfp*	Mauriello et al., 2009
EM440	*ΔfrzB frzCD-gfp*	This study
EM512	*ΔfrzA frzCD-gfp*	This study
EM515	*frzB-mCherry*	This study
EM605	*ΔfrzA ΔfrzB* $frzB^{\beta4\text{-}\beta5}$	This study
EM736	*ΔfrzA ΔfrzCD*	This study
EM742	*ΔfrzA ΔfrzB*	This study
EM743	*ΔfrzB ΔfrzCD*	This study
EM699	*ΔfrzB* $frzB^{\beta4\text{-}\beta5}$	This study
EM700	*ΔfrzA* $frzB^{\beta4\text{-}\beta5}$	This study
EM773	*ΔfrzA ΔfrzB frzCD-gfp*	This study
EM839	*ΔfrzA ΔfrzB* $frzB^{\beta4\text{-}\beta5}$ *frzCD-gfp*	This study
