# Supplementary material for: A divergent CheW confers plasticity to nucleoid-associated chemosensory arrays
Source: PLoS Genet. 2019 Dec 20;15(12):e1008533. doi: 10.1371/journal.pgen.1008533 (PMC6952110; doi:10.1371/journal.pgen.1008533)
Supplement: S2 Table — (PDF) [file pgen.1008533.s008.pdf]

Table S2. Plasmid list

Plasmid	Expression plasmid	Source
pEM411	pBJ113 with construct for *ΔfrzA frzCDΔ6-182*	This study
pEM412	pBJ113 with construct for *ΔfrzB frzCDΔ6-182*	This study
pEM463	pBJ113 with construct *ΔfrzA ΔfrzB* $frzB^{\beta4\text{-}\beta5}$ (Biomatik)	This study
pEM524	pBJ114 with construct *ΔfrzA ΔfrzB*	This study
pEM525	pBJ114 with construct *ΔfrzA* in DZ4480	This study
pEM526	pBJ114 with construct *ΔfrzB ΔfrzCD*	This study
pEM536	pBJ114 with construct *ΔfrzA* $frzB^{\beta4\text{-}\beta5}$	
pEM537	pBJ114 with construct *ΔfrzB* $frzB^{\beta4\text{-}\beta5}$	
pDPA20	pKY480 with construct for *frzCD-gfp*	Mauriello et al., 2009
pEM399	pBJ113 with construct for *frzB-mCherry*	This study
pETPhos *frzCD*	pETPhos with *frzCD* tagged with 6-his inducible with IPTG	Guzzo et al., 2015
pETPhos $frzE^{CheA}$	pETPhos with $frzE^{kinase}$ tagged with 6-his inducible with IPTG	Guzzo et al., 2015
pETPhos $frzCD^{c}$	pETPhos with $frzCD^{c}$ tagged with 6-his inducible with IPTG	Guzzo et al., 2015
pGEX(M) *frzA*	pGEX (M) with *frzA* tagged with GST	Guzzo et al., 2015
pEM464	pETPhos with $frzB^{\beta4\text{-}\beta5}$ tagged with 6-his inducible with IPTG (Biomatik)	This study
